# Retraction: Relationship between employees’ career maturity and career planning of edge computing and cloud collaboration from the perspective of organizational behavior

**DOI:** 10.1371/journal.pone.0292209

**Published:** 2023-10-10

**Authors:** The PLOS ONE Editors

After this article [1] was published, the first author (R. Zhang) requested retraction due to authorship concerns. Dr. Zhang alleged that she was unaware of the submission/article and did not contribute to the research reported therein. Of note, Dr. Zhang was listed as the corresponding author throughout the submission and peer review process, and pre-publication correspondence about the submission used the same email address that Dr. Zhang used in the post-publication inquiry. The second author (Y. Song) corroborated Dr. Zhang’s post-publication comments and stated that he used Dr. Zhang’s name and email address without permission. Dr. Song also informed PLOS that his affiliation is not correct as he has never been affiliated with Monash University and is not currently affiliated with an institution.

The *PLOS ONE* Editors also identified this article [1] as one of a series of submissions for which we have concerns about peer review integrity and similarities across articles. These concerns call into question the validity and provenance of the reported results. We regret that the issues were not identified prior to the article's publication.

In light of the above concerns, the *PLOS ONE* Editors retract this article.

RZ agreed with the retraction decision. YS did not agree with the retraction and apologized for the issues in the published article.
